# Supplementary material for: Identification of photoperiod-induced specific miRNAs in the adrenal glands of Sunite sheep (Ovis aries)
Source: Front Vet Sci. 2022 Jul 22;9:888207. doi: 10.3389/fvets.2022.888207 (PMC9354845; doi:10.3389/fvets.2022.888207)
Supplement: Supplementary file 1 [file Data_Sheet_1.ZIP › Supplementary file/miRDeep2 file.zip!/miRDeep2 file/Know_Structure/LP42A1/oar-mir-152.pdf]

miRBase precursor	: oar-mir-152
Total read count	: 22803
oar-miR-152 read count	: 22803
remaining reads	: 0

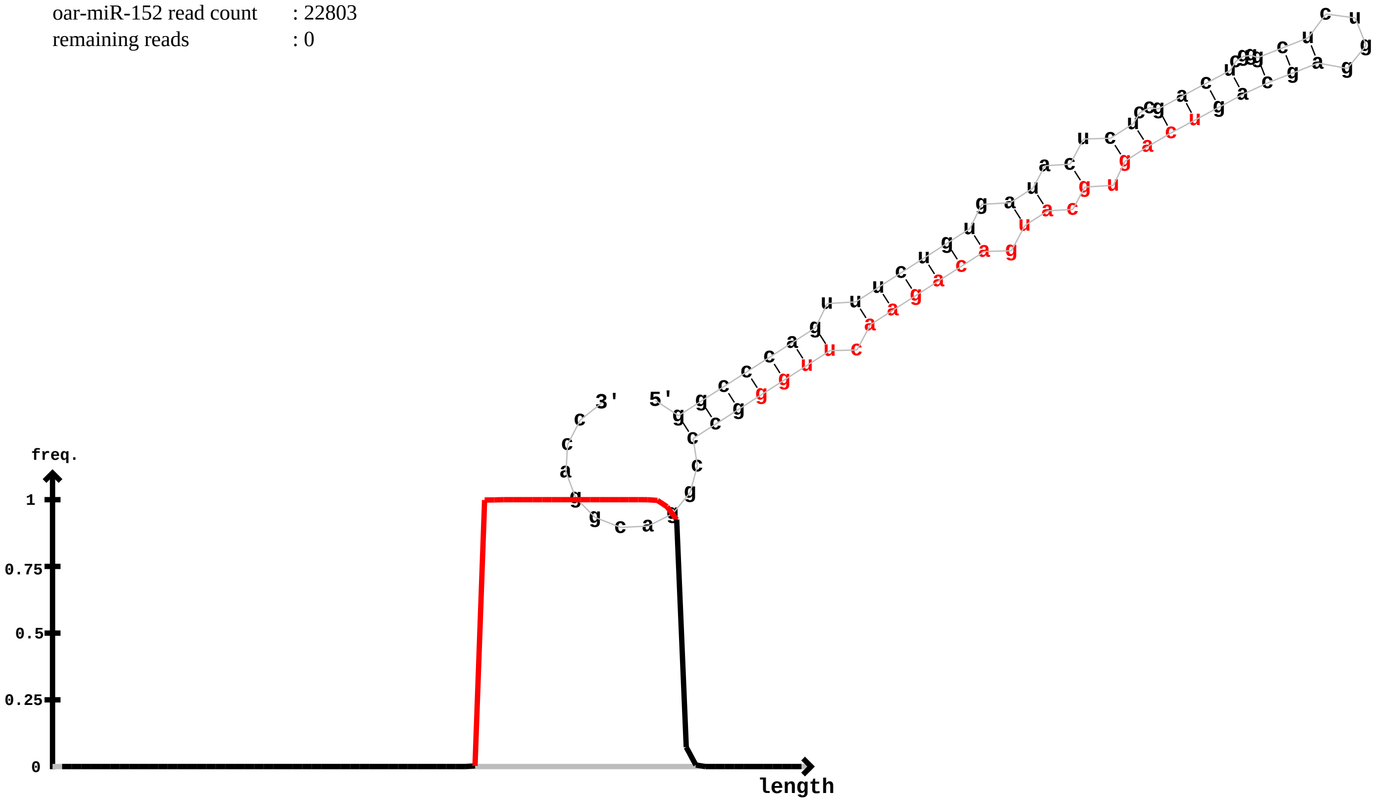

oar-miR-152

```
5'- ggcccaguuucugugauacucuccgacucgggcucuggagcagucagugcaugacagaacuugggcccggacggacc   -3'    exp
    (((((((.((((((.((.(.((..((((...(((....))))))))).).)).)))))).))))))).........
```

sequence	reads	mm	sample
....gucagugcaugacagaacuug..............	16	0	seq
....gucagugcaugacagaacuugg.............	39	0	seq
....gucagugcaugacagaacuuggg............	1	0	seq
....ucagugcaugacagaac.................	2	0	seq
....ucagugcauCacagaac.................	3	1	seq
....ucagugcaugacagaacu................	49	0	seq
....ucagugcauCacagaacu................	8	1	seq
....ucagugcaugacagaacuu...............	514	0	seq
....ucagugcauCacagaacuu...............	21	1	seq
....ucagugAaugacagaacuug..............	1	1	seq
....ucagugcaugacagaacuug..............	1081	0	seq
....ucagugcaugacagaacuugg.............	19389	0	seq
....ucagugcaugacagaacuuggg............	1516	0	seq
....ucagugcaugacagaacuugggc...........	132	0	seq
....ucagugcaugacagaacuugggccc.........	1	0	seq
....cagugcaugacagaacuu...............	2	0	seq
....cagugcaugacagaacuug..............	1	0	seq
....cagugcaugacagaacuugg.............	8	0	seq
....agugcaugacagaacuug..............	1	0	seq
....agugcaugacagaacuugg.............	12	0	seq
....agugcaugacagaacuugA.............	4	1	seq
....agugcaugacagaacuuggg............	2	0	seq
